# Clinical, biochemical, cellular and molecular characterization of mitochondrial DNA depletion syndrome due to novel mutations in the *MPV17* gene

**DOI:** 10.1038/ejhg.2013.112

**Published:** 2013-05-29

**Authors:** Johanna Uusimaa, Julie Evans, Conrad Smith, Anna Butterworth, Kate Craig, Neil Ashley, Chunyan Liao, Janet Carver, Alan Diot, Lorna Macleod, Iain Hargreaves, Abdulrahman Al-Hussaini, Eissa Faqeih, Ali Asery, Mohammed Al Balwi, Wafaa Eyaid, Areej Al-Sunaid, Deirdre Kelly, Indra van Mourik, Sarah Ball, Joanna Jarvis, Arundhati Mulay, Nedim Hadzic, Marianne Samyn, Alastair Baker, Shamima Rahman, Helen Stewart, Andrew AM Morris, Anneke Seller, Carl Fratter, Robert W Taylor, Joanna Poulton

**Affiliations:** 1 Nuffield Department of Obstetrics and Gynaecology, University of Oxford The Women's Centre, Oxford, UK; 2 Institute of Clinical Medicine/Paediatrics, University of Oulu, Clinical Research Center, Oulu University Hospital, Oulu, Finland; 3 Oxford Medical Genetics Laboratories, Oxford University Hospitals NHS Trust, Oxford, UK; 4 Wellcome Trust Centre for Mitochondrial Research, Newcastle University, UK; 5 Neurometabolic Unit, National Hospital for Neurology and Neurosurgery, London, UK; 6 Department of Pediatrics, King Fahad Medical City, Riyadh, Saudi Arabia; 7 Department of Pathology and Laboratory Medicine, King Abdulaziz Medical City, Riyadh, Saudi Arabia; 8 Department of Pediatrics, King Abdulaziz Medical City, King Saud Bin Abdulaziz University for Health & Science, Riyadh, Saudi Arabia; 9 Paediatric Liver Unit, Birmingham Children's Hospital, Birmingham, UK; 10 Department of Newborn Screening and Biochemical Genetics, Birmingham Children's Hospital, Birmingham, UK; 11 Clinical Genetics Unit, Birmingham Women's Hospital, Birmingham, UK; 12 Maternity Services, Manor Hospital, Walsall, UK; 13 Paediatric Liver Centre, King's College Hospital, London, UK; 14 Mitochondrial Research Group, UCL Institute of Child Health, London, UK; 15 Department of Clinical Genetics, Oxford University Hospitals NHS Trust, Oxford, UK; 16 Willink Biochemical Genetics Unit, St Mary's Hospital, Manchester, UK

**Keywords:** *MPV17*, mitochondrial DNA depletion, hepatocerebral disease, neonatal liver disease

## Abstract

Mitochondrial DNA (mtDNA) depletion syndromes (MDS) are severe autosomal recessive disorders associated with decreased mtDNA copy number in clinically affected tissues. The hepatocerebral form (mtDNA depletion in liver and brain) has been associated with mutations in the *POLG*, *PEO1* (Twinkle)*, DGUOK* and *MPV17* genes, the latter encoding a mitochondrial inner membrane protein of unknown function. The aims of this study were to clarify further the clinical, biochemical, cellular and molecular genetic features associated with MDS due to *MPV17* gene mutations. We identified 12 pathogenic mutations in the *MPV17* gene, of which 11 are novel, in 17 patients from 12 families. All patients manifested liver disease. Poor feeding, hypoglycaemia, raised serum lactate, hypotonia and faltering growth were common presenting features. mtDNA depletion in liver was demonstrated in all seven cases where liver tissue was available. Mosaic mtDNA depletion was found in primary fibroblasts by PicoGreen staining. These results confirm that *MPV17* mutations are an important cause of hepatocerebral mtDNA depletion syndrome, and provide the first demonstration of mosaic mtDNA depletion in human *MPV17* mutant fibroblast cultures. We found that a severe clinical phenotype was associated with profound tissue-specific mtDNA depletion in liver, and, in some cases, mosaic mtDNA depletion in fibroblasts.

## INTRODUCTION

During the last decade, an increasing number of nuclear genetic defects have been identified causing mitochondrial dysfunction either through the accumulation of mitochondrial DNA (mtDNA) deletions (multiple deletions) or through a reduction in mtDNA copy number causing mtDNA depletion syndromes (MDS), the latter of which are autosomal recessive diseases characterized by a severe, tissue-specific decrease of mtDNA copy number. At least 12 nuclear genes have been found to be involved in mtDNA maintenance, including *POLG*, *POLG2*, *PEO1*, *SLC25A4*, *TYMP*, *DGUOK*, *TK2*, *SUCLA2*, *SUCLG1*, *MPV17*, *OPA1* and *RRM2B* genes.^1^ MDS are caused by recessive defects in proteins directly involved in mtDNA replication or in proteins that affect the availability of deoxyribonucleoside triphosphates for mtDNA synthesis. Clinical presentations of MDS include early-onset hepatocerebral disease overlapping with Alpers-Huttenlocher syndrome (henceforth ‘Alpers'), isolated myopathy, encephalomyopathy and mitochondrial neurogastrointestinal encephalomyopathy syndrome. MDS are classified as myopathic, encephalomyopathic or hepatocerebral forms, of which the latter group has been associated with mutations in *POLG*, *PEO1* (Twinkle)*, DGUOK* and *MPV17* genes.^2^

The human *MPV17* gene is located on chromosome 2p21-23, comprising eight exons encoding 176 amino acids. It is expressed in human pancreas, kidney, muscle, liver, lung, placenta, brain and heart. *MPV17* encodes a mitochondrial inner membrane protein, which has a so far largely unknown role in mtDNA maintenance. Human *MPV17* is the orthologue of the mouse kidney disease gene, *Mpv17*. Loss of function has been shown to cause hepatocerebral MDS with oxidative phosphorylation failure and mtDNA depletion both in affected individuals and in *Mpv17*^−/−^ mice.^3^ To date, MDS caused by *MPV17* mutations has been reported in 32 patients with the clinical manifestations including early progressive liver failure, neurological abnormalities, hypoglycaemia and raised blood lactate.^4, 5, 6, 7, 8, 9, 10, 11, 12, 13, 14, 15^ Recently, *MPV17* mutations have also been associated with autosomal recessive adult-onset neuropathy and leukoencephalopathy with multiple mtDNA deletions in skeletal muscle.^16^ Thus, as for *POLG*, *RRM2B* and *TK2*, *MPV17* mutations can lead to recessive MDS or recessive multiple mtDNA deletion disorders.

The aim of this study was to clarify further the clinical, biochemical, cellular and molecular features associated with MDS due to *MPV17* gene mutations. We report 17 cases from 12 families with 11 novel *MPV17* mutations. All the patients presented with early-onset liver disease but only some patients manifested neurological dysfunction. Furthermore, we demonstrate that primary fibroblast cultures from patients with *MPV17* mutations may exhibit a mosaic mtDNA depletion.

## SUBJECTS AND METHODS

We studied 70 unrelated probands with suspected hepatocerebral MDS who had been referred to Mitochondrial Diagnostic Centres at Oxford, Newcastle or London for clinical assessment, histological, biochemical and/or molecular genetic analyses. This study was approved and performed under the ethical guidelines issued by each institution for clinical studies, with written informed consent obtained for all subjects.

### Molecular genetics

Total genomic DNA was isolated from blood leukocytes, skeletal muscle and liver samples by standard methods. Long-range PCR amplification of 13.8 kb mtDNA was undertaken to screen for mtDNA deletions in the available muscle and liver DNA samples, using primers previously described.^17^ mtDNA copy number relative to nDNA levels in muscle and/or liver DNA was estimated and compared with age-matched normal controls^18, 19^ by real-time quantitative PCR as described previously,^20^ except the nuclear probe was labelled with Vic at the 5′ end and the assays were carried out simultaneously using a PE7500 real-time PCR instrument (Applied Biosystems, Life Technologies, Carlsbad, CA, USA). mtDNA copy number <30% compared with age-matched controls was classed as mtDNA depletion, 30–50% as borderline low, and >50% as normal. The entire coding and flanking intronic regions of *MPV17* were amplified by PCR from genomic DNA and sequenced by fluorescent dideoxy sequencing (Applied Biosystems Big Dye Terminator v3.1 kit) and capillary electrophoresis (Applied Biosystems 3730). Results were compared with Genbank reference sequences NM_002437.4 and NG_008075.1, and mutations described in accordance with HGVS nomenclature guidelines. *MPV17* exon copy number (exons 1–8) was assessed by MLPA (kit P089-A1; MRC-Holland, Amsterdam, The Netherlands) in patients 16 and 17 (family 12) to confirm the deletion of multiple exons. Where available, parental blood DNA samples were analysed for the familial mutation(s) by sequencing of the appropriate exon, or by MLPA for family 12.

### Muscle histology, histochemistry and biochemistry

Tissue samples from muscle and liver were obtained according to standard procedures. Muscle and liver histology and histochemistry were performed by standard methods. The activities of mitochondrial respiratory chain enzyme complexes were determined from skeletal muscle and liver biopsy samples and cultured skin fibroblasts, as previously described.^21, 22^

### Characterization of the cellular mtDNA depletion phenotype by PicoGreen staining of fibroblasts

Cell cultures were analysed using the fluorescent stains PicoGreen (Molecular Probes, Eugene, OR, USA) (dsDNA intercalator) and tetramethylrhodamine methylester (TMRM; reflects mitochondrial membrane potential). Staining with PicoGreen is a simple, semi-quantitative method for visualizing mtDNA *in situ* within living cells as described.^23^ mtDNA nucleoids were further quantified using the IN Cell 1000 analyser (GE Healthcare Life Science, Little Chalfont, UK). Control and patients' fibroblasts were stained with PicoGreen and 30 nℳ TMRM as above in clear Optimem media (Gibco, Life Technologies). Raw images were acquired and binarized to quantify mtDNA nucleoids per cell. Nucleoids were defined as PicoGreen punctae present in the cytoplasm and colocalizing with mitochondria.

## RESULTS

### Analysis of the *MPV17* gene

**Figure 1:**
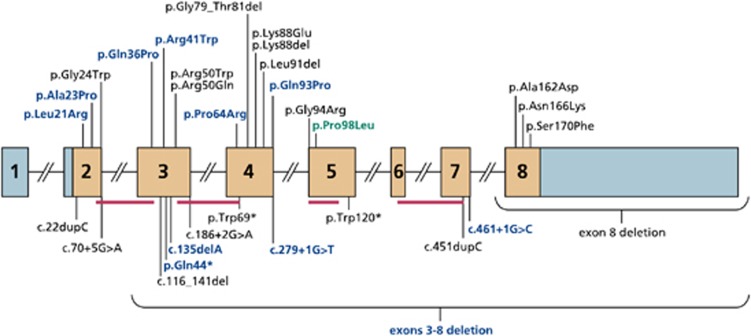
Mutations in the *MPV17* gene. Schematic representation of the *MPV17* gene, comprising eight exons of which exons 2–8 are coding (coding region coloured in orange); the positions of the 4 *α*-helical transmembrane spanning domains are indicated by red bars; missense/inframe deletion mutations are shown above the schematic and truncating/splicing mutations are shown below the schematic; novel mutations are indicated in blue, previously reported mutations also identified in this study in green and previously reported mutations not identified in this study in black.

**Figure 2:**
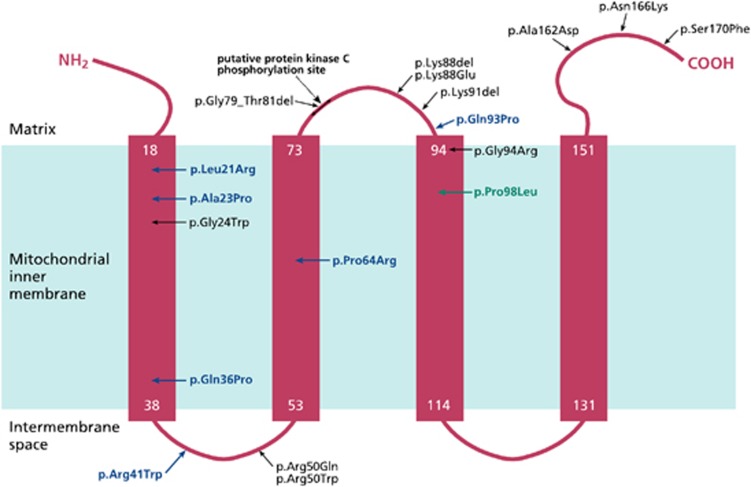
Location of missense mutations in the MPV17 protein. Schematic representation of the MPV17 protein, which is localized to the inner mitochondrial membrane; the 4 *α*-helical transmembrane spanning domains are indicated by red rectangles, and the number of the first and last amino acids of each of these domains is annotated in white; novel missense mutations are indicated in blue, previously reported missense mutations also identified in this study in green and previously reported missense/inframe deletion mutations not identified in this study in grey.

We identified pathogenic mutations in the *MPV17* gene in 12 out of 70 probands screened representing 17% of our undiagnosed cohort of children with suspected hepatocerebral MDS. Ten probands harboured homozygous mutations, and two had compound heterozygous mutations. A familial homozygous mutation was also identified in further affected siblings from families 4, 5, 9 and 12, resulting in a total of 17 patients from 12 families (Table 1). Eleven of the 12 mutations we identified are previously unpublished, and as such considered to be novel: c.62T>G (p.Leu21Arg), c.67G>C (p.Ala23Pro), c.107A>C (p.Gln36Pro), c.121C>T (p.Arg41Trp), c.130C>T (p.Gln44*), c.135delA (p.Glu45Aspfs*8), c.191C>G (p.Pro64Arg), c.278A>C (p.Gln93Pro), c.279+1G>T, c.461+1G>C and a deletion including exons 3–8 of *MPV17* (Figures 1 and 2).

Five of the 11 novel mutations are predicted to be protein truncating: c.279+1G>T and c.461+1G>C alter a consensus splice site; c.130C>T is a nonsense mutation (p.Gln44*); c.135delA results in a frameshift (p.Glu45Aspfs*8) and one mutation is a large deletion of exons 3–8. The remaining six novel mutations are missense changes, which are scattered throughout the protein (Figure 2), and alter amino acids, which are highly conserved across vertebrates (Supplementary Figure 1).

Homozygosity for the *MPV17* mutation in patients from families 1, 3–5, 7–10 and 12 was confirmed by parental testing, which demonstrated all parents to be heterozygous carriers. Parental samples were not available for family 11. For patients 2 (family 2) and 9 (family 6), at least one parent was found to be a heterozygous carrier in each case, thereby confirming compound heterozygosity.

### Molecular genetic studies on mtDNA from muscle and tissue samples

Molecular genetic analyses did not reveal any significant large-scale mtDNA rearrangements in any of the available muscle or liver DNA samples. mtDNA copy number was compared as previously^18, 19^ with controls matched for age but not ethnicity. Severe mtDNA depletion in liver was demonstrated for five out of seven cases (5–14% of control mtDNA) where liver tissue was available (patients 2, 3, 11, 14 and 16). Patient 9 had clear but less severe mtDNA depletion in liver (21% of controls), whereas mtDNA copy number was borderline low (40% of controls) in the remaining case (patient 4). Mean mtDNA copy number of all seven cases was 16%. In contrast, mtDNA copy number was highly variable across the six cases where muscle tissue was available (10–100% of controls, mean 46% Table 1).

### Clinical and laboratory features of patients with *MPV17* mutations

The 17 individuals in our cohort came from 12 families, 6 being male and 11 female, from different ethnic populations including Asian, Middle-Eastern and Caucasian families. All patients manifested with liver disease with abnormal liver function tests. Poor feeding, hypoglycaemia, raised plasma lactate, hypotonia and faltering growth were common preliminary features. In four cases, the growth failure was attributed to poor feeding, two poor weight gain in the presence of adequate food intake and one growth failure. Detailed clinical summaries for each patient are provided in Supplementary File 1.

Laboratory findings revealed hypoglycaemia, and patients with liver impairment had raised plasma levels of bilirubin, transaminases, GGT, ferritin, alpha fetoprotein and coagulopathy. Plasma lactate levels that were initially raised (ranging from 3 mmol/l to 21.4 mmol/l; normal range 0.7–2.1 mmol/l) usually decreased, corresponding to an obvious clinical improvement. CSF lactate values varied from normal to 5.1 mmol/l (normal range 1.1–2.2 mmol/l). Five of the 17 patients had at least one normal blood lactate or one normal CSF lactate. Plasma amino acids were increased including methionine, tyrosine and arginine (15-fold and 10-fold above the upper limit of the normal range, respectively), and a 2–3-fold increase of glutamine, alanine, serine, glycine or threonine.

Age at presentation ranged from birth to 5 years. Five patients (4, 9, 13, 14 and 17) have survived with current ages varying from 5 months to 11.5 years (mean age 4.4 years). The median age at death was 13 months (range from 3 months to 4.3 years). Two patients (6 and 9) underwent liver transplantation. Patient 6 received a liver transplant at the age of 9 months, but died due to neurological and renal manifestations at the age of 2.5 years. Conversely, patient 9 received a liver transplant at the age of 3 years and is currently 11.5 years old manifesting with progressive demyelinating peripheral neuropathy, hypoparathyroidism and severe growth hormone deficiency. Where studied, liver ultrasound showed an enlarged echogenic liver (patients 1, 7 and 12), a distended gall bladder with biliary sludge (patients 1 and 8), nodular/heterogeneous liver parenchyma (patients 4, 5 and 8) and ascites in the abdomen and pelvis (patients 5 and 12). Brain MRI scans revealed global cerebral and cerebellar atrophy (patient 6), brain infarction (patients 6 and 7) and white matter changes consistent with metabolic leucodystrophy in five patients (patients 1, 4, 5, 6 and 10) from our cohort. Neuropathy was a feature of family 4, with corneal scarring likely due to sensory deficit, as described in Navajo familial neurogenic arthropathy.^24^

### Histopathological features

The histological assessment of a diagnostic muscle biopsy was performed in six patients. Histopathological changes varied from normal findings (patients 2, 4 and 5) to fatty infiltration (patients 7 and 9), to a generalized fibre atrophy with small angular fibres (patient 16).

Liver histology was available from 13 of the 17 patients. Histopathological features included: fatty infiltration (patients 2, 4, 6, 7, 9, 10, 13, 14 and 16), fibrosis/cirrhosis (patients 2, 4, 5, 10 and 14), severe panlobular loss of hepatocytes and stromal collapse (patient 3), giant cell hepatitis with haemorrhagic necrosis (patient 6), non-specific changes (patient 11), cholestasis (patients 14 and 16), patchy hepatocellular oncocytosis and single-cell necrosis (patient 15), abundant mitochondria with mild pleomorphism (patient 9), marked distension of hepatocytes and few periportal glycogen deposits (patient 10) and mild iron deposition in Kupffer cells (patient 13).

### Biochemical and histochemical analyses

Table 1 Muscle tissue, liver tissue and/or skin fibroblasts were available for the assessment of mitochondrial respiratory chain function from 7 out of 12 probands. Respiratory chain analysis in muscle at or soon after initial clinical presentation suggested a combined deficiency of respiratory chain complex activities in four patients (7, 9, 10 and 15), whereas the enzyme activities were normal in muscle samples from patients 2, 3 and 4 as well as in liver from patient 4 (who had the highest mtDNA copy number) and fibroblasts from patient 3. Unfortunately, biochemical analyses of liver from patients 3, 11 and 15 with mosaic mtDNA depletion in fibroblasts were not available. Sequential COX-SDH histochemistry revealed a severe, near-global COX deficiency in the liver from patient 2. Initial muscle histology at the age of 28 months from patient 9 was within normal limits, concurrent respiratory enzyme assays showing a borderline complex I deficiency. A repeat muscle biopsy from patient 9 at 3 years revealed slightly decreased respiratory chain activities of both complex I and complex IV (cytochrome c oxidase; 82% of control values). Complex IV activity was 27% of control in the explanted liver tissue. These findings are also summarized in Table 1.

### Characterization of the cellular mtDNA depletion phenotype by PicoGreen staining of fibroblasts

**Figure 3:**
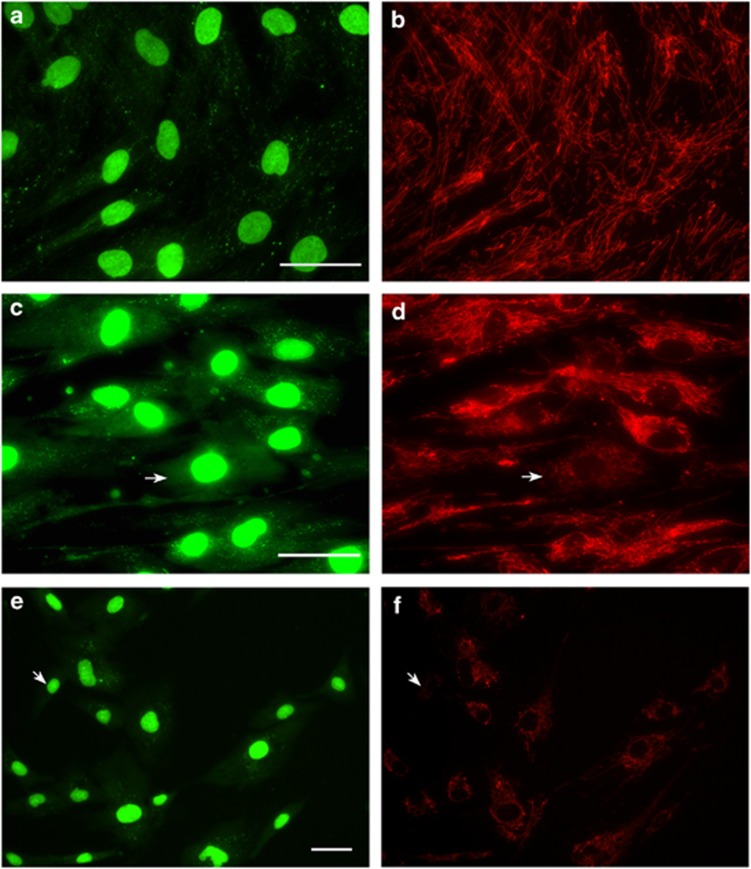
PicoGreen staining of fibroblast cultures. Healthy control fibroblasts exhibit typical bright punctate nucleoid staining when stained with the fluorescent DNA dye PicoGreen (**a**). Co-staining these cells with the potential sensitive mitochondrial stain, TMRM demonstrated a normal polarized mitochondrial network in all cells (**b**). Fibroblasts from patient 3 mostly exhibit typical bright nucleoids staining, but some cells (arrow) lack puncta (green in on-line version) because they are depleted of mtDNA, termed mosaic mtDNA depletion (**c**). Co-staining of these cells with TMRM demonstrates that some of the mtDNA-depleted cells have a depolarized mitochondrial network (**d**). Fibroblasts grown from patient 17 umbilical cord also show mosaic mtDNA depletion (**e**), most cells having normal TMRM signal (**f**) but one depolarized cell is shown (arrow) consistent with mosaic mtDNA depletion. Scale bars=50 μm.

Fibroblast cultures were available from 8 of the 17 patients in our cohort. mtDNA was visualized in these cultures using PicoGreen fluorescence microscopy and the membrane potential-dependent mitochondrial probe, TMRM. PicoGreen staining provides a quantitative measure of mtDNA content in a cell, and is more sensitive to small localized depletion than real-time PCR. mtDNA is arranged into punctate DNA/protein structures, termed nucleoids *in vivo*, consisting of several mtDNA genomes complexed to nucleoid proteins. Mosaic mtDNA depletion was clearly observed in fibroblasts from four *MPV17* patients (patients 3, 11, 15 and 17; Figures 3c and e and Supplementary Information 2). In two patients, intermediate mtDNA depletion was observed with fibroblast mtDNA staining being generally paler (patients 4 and 8) whereas PicoGreen staining was normal in fibroblasts from patients 2 and 9. Cells showing the most marked mtDNA depletion exhibited a decreased mitochondrial membrane potential following TMRM staining. This was reflected by real-time PCR (Supplementary Information 2), which showed that the average mtDNA copy number in the cell lines with mosaic depletion was 70% (range 35–100%) of expected compared with 89% (range 43–150%) in the other patient lines (four controls, mean 100%, range 70–136%). Real-time PCR was supported by quantitation of nucleoid numbers using IN Cell 1000 analyser (Supplementary Information 2).

## DISCUSSION

We have performed clinical, biochemical, immunocytochemical and molecular genetic studies of 17 patients with MDS associated with *MPV17* mutations, demonstrating a loose relationship between the clinical phenotype and mutational genotype, with patients with the most severe mtDNA depletion in liver tending to present and die at an earlier age. We have also demonstrated a mosaic mtDNA depletion in fibroblasts from some of the more severely affected patients.

Previously, *MPV17* mutations have been reported in 32 patients with the hepatocerebral form of MDS in which the most common clinical manifestations included early progressive liver failure, hypoglycaemia and raised plasma lactate concentrations.^4, 5, 6, 7, 8, 9, 10, 11, 12, 13, 14, 15^ Some of these patients manifested with neuromuscular abnormalities including hypotonia, nystagmus, encephalopathy, developmental delay/mental retardation, microcephaly, seizures, myoclonus, myopathy, ataxia and/or peripheral neuropathy (Navajo neurohepatopathy). White matter changes and abnormalities within the reticular formation of the lower brain stem and within the reticulospinal tracts at the cervicocranial junction on MR imaging have been reported.^13, 15^

### Clinical findings and differential diagnoses

All our 17 patients presented with liver disease; other common features included raised serum lactate concentrations, hypotonia and faltering growth. Rarer features included endocrine abnormalities (hypoparathyroidism in patient 9, pituitary dysfunction in patient 12 and hypothyroidism in patient 15), retinal pigmentation (patients 10 and 13), corneal scarring^24^ (patient 5) and progressive demyelinating peripheral neuropathy (patients 4 and 9).^7, 11^ In addition, three patients were found with minor dysmorphic features (patients 12, 15 and 16), which have not been described previously. Thus, the clinical findings in our cohort of MDS patients with *MPV17* mutations are broadly similar to those previously reported.

The infantile phenotypes of hepatocerebral forms of MDS associated with mutations in *POLG*, *PEO1* (Twinkle), *DGUOK* or *MPV17* have similarities, presenting with liver failure along with various neurological and endocrinological manifestations. Significant hypoglycaemia, that is glucose levels that are more difficult to maintain with oral supplementation than would be expected from the degree of liver failure, was commonly present in patients with *MPV17* mutations as previously reported.^4^ We also observed raised levels of tyrosine in blood and/or urine in three patients, as can occur in patients with *DGUOK* mutations.^24^ We have also noted a tendency for the liver failure to precede severe neurological problems, whereas in our experience the sequence tends to be the other way round in patients with classical Alpers syndrome due to *POLG* mutations: in only 1 out of 24 of our series did liver dysfunction clearly precede neurological involvement.^20^ For this reason, patient 6 underwent a liver transplant at 15 months of age, but died 15 months later, following neurological and renal deterioration. In summary, presentations of patients with *MPV17* mutations were most like those of patients with *DGUOK*-associated MDS, who typically have neonatal liver failure and hypotonia.^25^

### MPV17 mutations

Twelve different mutations were identified in our cohort of 17 patients (12 probands), including 11 novel mutations (6 missense, 5 truncating) in the *MPV17* gene (Figure 1). This adds significantly to the 21 previously reported mutations.^4, 5, 6, 7, 8, 9, 10, 11, 12, 13, 14, 15, 16^ The seven missense mutations (six novel and one previously reported) identified in our cohort are scattered throughout the protein (Figure 2), with no significant clustering. This is in contrast to El-Hattab *et al*,^11^ who reported clustering of missense mutations in the region of the putative protein kinase C phosphorylation site.

The majority of patients in our cohort presented in the first few weeks of life and died before 2 years of age (although three patients currently aged 5–14 months are living and so their clinical course cannot be predicted), and these patients had either truncating or missense mutations in *MPV17* that presumably led to complete loss of functional protein. However, patients from families 4 and 6 (patients 4, 5, 6 and 9) appear to have a milder disease with better prognosis. These patients are/were either homozygous or compound heterozygous for missense mutations, namely p.Arg41Trp, p.Pro64Arg and p.Pro98Leu, suggesting that these mutant MPV17 proteins retain some residual function. This is similar to the findings of Karadimas *et al*,^5^ who reported patients homozygous for p.Arg50Gln or compound heterozygous for p.Gly94Arg and p.Pro98Leu, with a relatively mild form of hepatocerebral MDS, namely Navajo neurohepatopathy. It is interesting to note that both p.Arg41Trp and p.Arg50Gln are located in the same protein loop in the intermembrane space (Figure 2).

As a result of identifying pathogenic *MPV17* mutations, prenatal diagnosis and/or preimplantation genetic diagnosis is now available to these 12 families. Indeed, the parents of patient 16 opted to have amniocentesis and *MPV17* analysis in two subsequent pregnancies, who were accurately identified as an unaffected carrier and an affected girl (patient 17).

### Mitochondrial respiratory chain analyses and mtDNA depletion in tissue samples

Respiratory chain deficiency and mtDNA depletion are usually present in the muscle of MDS patients, but not in all patients. In cases with isolated liver involvement, respiratory chain enzyme activity and mtDNA copy number have been normal in muscle and decreased only in liver tissue. For example, many patients with autosomal recessive mutations in the *POLG* gene causing Alpers syndrome (intractable epilepsy, hepatopathy) have normal respiratory chain enzyme activity and mtDNA copy number in skeletal muscle.^20, 26^ The characterization of tissues from previously described patients with *MPV17* mutations has revealed a severe mtDNA depletion that was most pronounced in liver, followed by a less severe, but still significant depletion in skeletal muscle.^6, 15^ In our cohort, respiratory chain analysis in muscle revealed a combined deficiency of respiratory chain complexes in four out of seven muscle biopsy samples available for biochemical studies, and mtDNA depletion was identified in three out of eight muscle samples with borderline low copy number in two samples. Therefore, muscle from three out of eight patients showed no evidence of mtDNA depletion. Interestingly, there was no clear correlation between the level of mtDNA depletion in muscle, disease severity or mutation location.

As predicted, respiratory chain enzyme activities and mtDNA copy number in liver tissue correlated more closely with disease severity. Liver was available for mtDNA copy number analysis in seven patients. Severe mtDNA depletion in liver (<20% compared with age-matched controls) was associated with early onset and severe disease course (Table 1). In addition, when measured in both tissues (five patients), the mtDNA content was significantly less in the liver than in the muscle (*P*=0.04, one-tailed paired sample *T*-test). Both of these features are also apparent in patients with Alpers syndrome due to *POLG* mutations.^20^ Although liver respiratory chain enzyme activities or histochemistry were only available for three patients, this also showed some correlation with disease severity, as the only sample with normal activity was from patient 4 with the mildest disease.

### PicoGreen stainings of fibroblasts

We identified mosaic mtDNA depletion in fibroblasts by PicoGreen staining in three cases (patients 3, 11 and 15) with an early-onset liver disease (birth–2.5 months), two of whom had severe mtDNA depletion in liver (5% in patient 3 and 11% in patient 11); fibroblasts grown from the umbilical cord of patient 17 (now 5 months old and deteriorating rapidly with liver failure, and whose older sibling, patient 16, was severely affected and had severe liver mtDNA depletion) also showed mosaic mtDNA depletion. In four other patients (2, 4, 8 and 9) from whom fibroblasts were available, there was no clear evidence of mosaic mtDNA depletion even after extensive passaging, and these patients had more variable onset (birth–5 years) and mtDNA copy number in liver (14–40% of controls). Muscle mtDNA copy number was highly variable both in patients with mosaic mtDNA depletion (25–100% of controls) and in patients without mosaic mtDNA depletion (46–80% of controls). Thus, mosaic mtDNA depletion was more apparent in patients with severe clinical phenotype and low mtDNA content in liver, but did not appear to correlate with muscle mtDNA content. A similar association between the cellular phenotype and severity of the clinical phenotype has previously been seen in patients with the most severe *POLG* mutations.^20, 23^ Mosaic mtDNA depletion has also been detected in serum-deprived mouse embryonic fibroblasts lacking MPV17 protein, using antiDNA antibodies,^3^ but has not previously been observed in patients with *MPV17* mutations. Finding mosaic depletion in fibroblasts, post mortem may provide a useful clue to aetiology in cases where fibroblasts are the only material available for diagnosis.^27^

## CONCLUSION

Our data confirm previous reports describing *MPV17* mutations as an important cause of mtDNA maintenance disorders, specifically hepatocerebral MDS. We describe 17 patients with 11 novel *MPV17* mutations and provide the first description of recessive *MPV17* mutations associated with mosaic mtDNA depletion in fibroblasts. We present evidence that *MPV17* mutations lead to tissue selective impairment of mtDNA replication and to a mosaic defect pattern in fibroblasts, which is associated at least in some patients with the severity of clinical phenotype.

## Figures and Tables

**Table 1:** Clinical, biochemical, cellular and molecular findings in 17 patients with *MPV17* mutations

*Family*	*Patient, sex*	*Consanguinity (family history)*	*Clinical information*	*Age at onset*	*Age at referral*	*Deceased/alive, age at death/current age*	*MPV17 mutations*	*mtDNA copy number in liver*	*mtDNA copy number in muscle*	*PicoGreen analysis of fibroblasts*	*Muscle/liver histology*	*Muscle/liver/fibroblasts respiratory chain activities or histochemistry*
1	1, F	Y (N)	Hypotonia, progressive liver disease, coagulopathy, hypoglycaemia, developmental delay, raised blood lactate	4.5 mo	5 mo	Decd, 6.5 mo	c.62T>G (p.Leu21Arg) hom	ND	ND	ND	ND	ND
2	2, F	N (N)	Poor feeding, FG, progressive liver disease, hypoglycaemia, raised blood lactate, delayed development, vomiting	Birth	2 mo	Decd, 12 mo	c.67G>C (p.Ala23Pro) & c.135delA (p.Glu45Aspfs*8)	14%	51%	Normal	Mus: Normal; Liv: micro-and macro-vesicular change in most hepatocytes, fibrosis, inflammation	Mus: Normal; Liv: CIV↓
3	3, F	Y (Y^a^)	IUGR, feeding problems, hypoglycaemia, raised tyrosine within days of birth, raised blood lactate, FG, hypotonia, progressive liver failure, encephalopathy	Birth	2 mo	Decd, 4 mo	c.107A>C (p.Gln36Pro) hom	5%	100%	Mosaic depletion	Liv: severe panlobular loss of hepatocytes and stromal collapse	Mus: Normal; Fib: Normal
4	4, F	Y (Y, sibs P5 & P6)	Progressive neurological deterioration with peripheral neuropathy, progressive liver disease	5 yr	5 yr	Alive, 8.5 yr	c.121C>T (p.Arg41Trp) hom	40%	46%	Normal/minor changes	Mus: Normal; Liv: microvesicular steatosis and focal fibrosis	Mus: Normal; Liv: Normal
	5, F	Y (Y, sibs P4 & P6)	Progressive liver failure, FG, corneal scarring , progressive neurological deterioration	Birth	9 mo	Decd, 4 yr 3 mo	c.121C>T (p.Arg41Trp) hom	ND	38%	N.D.	Mus: Normal Liv: cirrhosis	ND
	6, M	Y (Y, sibs P4 & P5)	Progressive liver failure, encephalopathy, liver transplant at 15 mo, died 15 mo later following neurological and renal deterioration.	Birth	9 mo	Decd, 2 yr 6 mo	ND	ND	ND	ND	Liv: Giant cell hepatitis, haemorrhagic necrosis, focal fatty change	ND
5	7, M	Y (Y^b^, sib P8)	Poor feeding, hypoglycaemia, FG, raised blood lactate, encephalopathy, progressive liver failure	3.5 mo	3.5 mo	Decd, 4 mo	c.130C>T (p.Gln44*) hom	ND	ND	ND	Mus: Fatty infiltration; Liv: fatty infiltration	Mus: CI ↓, CII+III ↓, CIV ↓
	8, F	Y (Y^b^, sib P7)	Tachypnoea, hypoglycaemia, raised blood lactate within days of birth, poor weight gain, progressive liver failure	Birth	Birth	Decd, 7.5 mo	c.130C>T (p.Gln44*) hom	ND	ND	Normal/minor changes	ND	ND
6	9, M	N (N)	FG, liver disease, liver transplant at 3 yr, progressive demyelinating peripheral neuropathy, hypoparathyroidism, severe growth hormone deficiency, gastrostomy	5 mo	5 mo	Alive, 11.5 yr	c.191C>G (p.Pro64Arg) & c.293C>T (p.Pro98Leu)	21%	80%	Normal	Mus: minor fatty changes; Liv: steatosis, abundant mitochondria with mild pleomorphism	Mus: CI ↓, CIV↓ Liv: CIV ↓
7	10, M	Y (N)	Neonatal jaundice, FG, hypotonia, microcephaly, progressive liver disease, raised blood lactate, retinal pigmentation	2 mo	2 mo	Decd, 5 mo	c.278A>C (p.Gln93Pro) hom	ND	ND	ND	Liv: distension of hepatocytes, few periportal glycogen nuclei, fibrosis, microvesicular steatosis and portal inflammation.	ND
8	11, F	Y (Y^c^)	Poor feeding, mild hypotonia, jaundice, liver dysfunction, coagulopathy, raised blood lactate	2 mo	3.5 mo	Decd, <16 mo	c.278A>C (p.Gln93Pro) hom	11%	ND	Mosaic depletion	Liv: cholestasis with scattered giant cell hepatocytes and extensive portal - portal bridging fibrosis	ND
9	12, F	Y (Y, sib P13)	Poor feeding, FG, hypotonia, subtle facial dysmorphism, hypoglycaemia, cholestatic jaundice, central hypocortisolism, progressive liver disease	2 mo	5 mo	Decd, 8 mo	c.278A>C (p.Gln93Pro) hom	ND	10%	ND	ND	Mus: CI ↓, CIII ↓, CIV ↓
	13, F	Y (Y, sib P12)	Neonatal jaundice, FG, hypotonia, recurrent vomiting, progressive liver disease, raised blood lactate, retinal pigmentation	Birth	5 mo	Alive, 6 mo	c.278A>C (p.Gln93Pro) hom	ND	ND	ND	Liv: large droplet steatosis and microsteatosis, mild iron deposition in Kupffer cells	ND
10	14, M	Y (Y^d^)	FG, dev delay, hypotonia, myopathy, liver disease, cholestasis; raised blood lactate	4 mo	5 mo	Alive, 14 mo	c.279+1G>T hom	5%	ND	ND	Liv: micro- and macro-vesicular steatosis with canalicular cholestasis, portal fibrosis and septae formation	ND
11	15, F	Y (N)	Conjugated jaundice, hypothyroidism, hypotonia, FG, developmental delay and dysmorphism	2.5 mo	13 mo	Decd, 3 yr	c.461+1G>C hom	ND	25%	Mosaic depletion	Liv: Patchy hepatocellular oncocytosis and single-cell necrosis	Mus: CI ↓, CII+III ↓, CIV ↓
12	16, M	Y (N)	Poor weight gain, hypoglycaemia, FG, hypotonia, subtle dysmorphic features, progressive liver failure, encephalomyopathy	2 mo	3 mo	Decd, 4 mo	Del exons 3–8 hom	14%	17%	ND	Mus: Generalized fibre atrophy, small angular fibres; Liv: cholestasis and steatosis	ND
	17, F	Y(Y, sib P16)	Raised *γ*GT at 6 weeks, progressive liver failure from 4 mo with rapid deterioration	Birth	Birth	Alive, 5 mo	Del exons 3-8 hom	ND	ND	Mosaic depletion	ND	ND

Abbreviations: CI, CII, CIII, CIV, mitochondrial respiratory chain enzyme complexes I, II, III and IV, respectively; Decd, deceased; F, female; FG, faltering growth; Fib, fibroblasts; *γ*GT, gamma–glutamyltransferase; IUGR, intrauterine growth retardation; Liv, liver; M, male; mo, months; Mus, muscle; N, no; ND, not determined; Y, yes; yr, years.

a Two relatives died in infancy due to liver disease (the mother's sister's two children whose parents were also consanguineous).

b Liver disease in two family members, two premature deaths in infancy of two other family members.

c Two cousins died with liver disease.

d Two previous siblings died in infancy with similar presentation.
