# Supplementary material for: Clinical, biochemical, cellular and molecular characterization of mitochondrial DNA depletion syndrome due to novel mutations in the MPV17 gene
Source: Eur J Hum Genet. 2013 May 29;22(2):184–91. doi: 10.1038/ejhg.2013.112 (PMC3895632; doi:10.1038/ejhg.2013.112)
Supplement: Supplementary Files 1 [file ejhg2013112x1.doc]

**Supplementary Figure 1: Evolutionary conservation of mutated amino acids detected in our *MPV17* patient cohort**

Human MA-LWRAYQRALAAHPWKVQV**L**T**A**GSLMGLGDIISQ**Q**LVER**R**GLQE-HQRGRTLTMVSLG 58

Chimp MA-LWRAYQRALAAHPWKVQV**L**T**A**GSLMGLGDIISQ**Q**LVER**R**GLQE-HQRGRTLTMVSLG

Macaque MA-LWRAYQRALAAHPWKVQV**L**T**A**GSLMGLGDIISQ**Q**LVER**R**GLQE-HQRGRTLTMMSLG

Rat MA-LWRAYQRALAAHPWKVQV**L**T**A**GSLMGLGDIISQ**Q**LVER**R**GLQQ-HQTGRTLTMASLG

Mouse MA-LWRAYQRALAAHPWKVQV**L**T**A**GSLMGVGDMISQ**Q**LVER**R**GLQQ-HQAGRTLTMVSLG

Cow MA-LWRAYQRALTAHPWKVQV**L**T**A**GSLMGLGDVISQ**Q**LVER**R**GLQA-HQAGRTLTMASLG

Cat MA-LWRAYQRALTVHPWKVQV**L**T**A**GSLMGLGDIISQ**Q**LVER**R**GLRE-HQTGRTLTMVSVG

Frog MAGLWRAYQRLLGAHPWKVQIVT**A**GSLVGVGDVISQ**Q**LLERKGLKG-HSIERTVKMMGIG

Zebrafish MAGLWRSYQALMAKHPWKVQIIT**A**GSLVGVGDVISQ**Q**LIER**R**GLAN-HNARRTAKMMSIG

Tetraodon MAGLWRAYQSLMSRYPWTVQIVT**A**GSLVGVGDVISQ**Q**LIER**R**GVAH-HNMRRTAKMMSIG

Fruitfly ----------MKRLKAYLKDGINVAAVMCLGDTISQFFFDKKSLDE-WDAGRTLRFGIVG

*S.cerevisiae* -MKLLHLYEASLKRRPKTTNAIMTGALFGIGDVSAQLLFPTSKVNKGYDYKRTARAVIYG

Human CGFVG**P**VVGGWYKVLDRFIPGTTK-----VDALKKMLLD**Q**GGFA**P**CFLGCFLPLVGALNG 113

Chimp CGFVG**P**VVGGWYKVLDRFIPGTTK-----VDALKKMLLD**Q**GGFA**P**CFLGCFLPLVGALNG

Macaque CGFVG**P**VVGGWYKVLDRFIPGTTK-----VDALKKMMLD**Q**GGFA**P**CFLGCFLPLVGALNG

Rat CGFVG**P**VVGGWYRVLDHLIPGTTK-----VNALKKMLLD**Q**GGFA**P**CFLGCFLPLVGVLNG

Mouse CGFVG**P**VVGGWYKVLDHLIPGTTK-----VHALKKMLLD**Q**GGFA**P**CFLGCFLPLVGILNG

Cow CGFVG**P**VVGGWYRVLDRLIPGTTK-----VDALKKMLLD**Q**GGFA**P**CFLGCFLPLVGTLNG

Cat CGFVG**P**VVGGWYRVLDRLVPGTTK-----VDALKKMLLD**Q**GGFA**P**CFLGCFLPLVGALNG

Frog FCFVG**P**VVGGWYKILDRIIPGSGK-----PVALKKMLLD**Q**VAFA**P**CFLGCFLSIASALNG

Zebrafish FFFVG**P**VVGGWYKVLDKLVTGGTK-----SAALKKMLVD**Q**VGFA**P**CFLGAFLGITGTLNG

Tetraodon FFFVG**P**VIGSWYKVLDRLVVGGSR-----SAAMKKMLVD**Q**LCFA**P**CFLAAFFCVSGAVNG

Fruitfly LVFVG**P**TLRRWYHFLESRVPKTYSP---MRRGVTKMLVD**Q**TLFA**P**PFTMAMSFLVPLSNG

*S.cerevisiae* SLIFSFIGDKWYKILNNKIYMRNRPQYHWSNMVLRVAVD**Q**LAFA**P**LGLPFYFTCMSIMEG

Human LSAQDNWAKLQRDYPDALITNYYLWPAVQLANFYLVPLHYRLAVVQCVAVIWNSYLSWKA 173

Chimp LPAQDNWAKLQRDYPDALITNYYLWPAVQLANFYLVPLHYRLAVVQCVAVIWNSYLSWKS

Macaque LSAKDNWAKLQRDYPDALITNYYLWPAVQLANFYLVPLHYRLAVVQCVAVIWNSYLSWKA

Rat MSAQDNWAKLKRDYPDALITNYYLWPAVQLANFYLVPLHYRLAVVQCVAVVWNSYLSWKA

Mouse MSAQDNWAKLKRDYPDALITNYYLWPAVQLANFYLVPLHYRLAVVQCVAIVWNSYLSWKA

Cow LSAQDNWAKLQRDFPDALITNYYLWPAVQLANFYLVPLHYRLAVVQCVAVIWNSYLSWKA

Cat LSAQDNWAKLRQDYPDALITNYYLWPAVQLANFYLVPLHYRLAVVQCVAVLWNSYLSWKA

Frog LSGEQIWGKLKRDYKDALITNYYIWPAVQVANFYFIPLYHRLAVVQFVAIIWNSYLSWKA Zebrafish LTVEENVAKLQRDYTDALISNYYLWPPVQIANFYFIPLHHRLAVVQIVAVVWNSYLSWKA

Tetraodon LTVEDNLGKLQRDYADALISNYYLWPPVQIANFYFVPLHHRLAVVQVVAVGWNSYLTWKA

Fruitfly EPIDRIRQRILDSYLSILVRNYMLWPAAQMLNFRFVPLGYQVLYAQFIALVWNCYLSMIL

*S.cerevisiae* RSFDVAKLKIKEQWWPTLLTNWAVWPLFQAINFSVVPLQHRLLAVNVVAIFWNTYLSYKN

Human HRL--------------- 176

Chimp TSALSLPHSIVSTLQ---

Macaque HRL---------------

Rat HQL---------------

Mouse HQF---------------

Cow HRL---------------

Cat HRL---------------

Frog NKS---------------

Zebrafish NKM---------------

Tetraodon NKM---------------

Fruitfly NS----------------

*S.cerevisiae* SKVMEKDKVPVHYPPVVE

**CLUSTAL W (1.82) multiple sequence alignment of *MPV17* sequences/homologues.** The locations of amino acids altered by the 6 novel missense substitutions (p.Leu21Arg, p.Ala23Pro, p.Gln36Pro, p.Arg41Trp, p.Pro64Arg & p.Gln93Pro) are highlighted in blue. The location of p.Pro98Leu, which has been previously reported by Karadimas *et al.* 2006^4^ and was also identified in this cohort, is highlighted in green.
