# Supplementary material for: Clinical, biochemical, cellular and molecular characterization of mitochondrial DNA depletion syndrome due to novel mutations in the MPV17 gene
Source: Eur J Hum Genet. 2013 May 29;22(2):184–91. doi: 10.1038/ejhg.2013.112 (PMC3895632; doi:10.1038/ejhg.2013.112)
Supplement: Supplementary Figures 1 [file ejhg2013112x2.doc]

**Supplementary File 1**

**Case reports of patients with *MPV17* mutations.**

**Family 1*. Patient 1*** was a girl, the first baby born to first cousin parents, who presented at the age of 4.5 months with jaundice, hypotonia, hepatomegaly and hypoglycaemia. At presentation, laboratory investigations revealed abnormal liver function tests. One month after presentation coagulopathy and raised blood lactates were noticed. Ultrasound scan of her abdomen revealed mild hepatomegaly. Brain MRI showed abnormal signal intensity within the inferior cerebellar peduncles bilaterally. At the age of 5 months she presented with persistent hypoglycaemia and mild coagulopathy, but jaundice resolved and liver function tests were normal. At 6 months of age laboratory tests showed that she was developing a progressive coagulopathy with impaired liver function. Clinically, she developed jaundice again with increasing lethargy. She died at the age of 6.5 months, 2 months after admission, due to progressive liver failure.

**Family 2*. Patient 2*** was a girl who was born by a normal delivery at term following a normal pregnancy. She presented at the age of 4 days with poor feeding, vomiting and weight loss. She was jaundiced but was otherwise normal on clinical examination. The patient was admitted to hospital and found to have abnormal liver function and recurrent episodes of hypoglycaemia. The latter occurred after fasting for as little as 30 minutes, necessitating continuous feeding for several months (initially through a nasogastric tube and later via a gastrostomy). The plasma 3-hydroxybutyrate concentration was low relative to the free fatty acid concentration (0.4 mmol/l and 2.04 mmol/l, respectively). Blood acylcarnitine analysis and urine organic acid analysis were unremarkable apart from raised tyrosine in urine. Plasma lactate concentrations were consistently raised together with CSF lactate. She had increased primary bile acids in urine, consistent with cholestasis, but no abnormal conjugates or bile acid intermediates. There was generalised aminoaciduria. A muscle biopsy was taken from quadriceps femoris at 2 months and a liver biopsy was taken at 4 months of age. The patient became better able to tolerate fasting, but vomiting was a persistent problem despite continuous feeding, thickening her feeds and treating with domperidone and omeprazole. Feeding through a gastro-jejunal tube led to some improvement. Weight gain was initially very slow; it improved after 10 weeks of age but always remained below the 0.4^th^ centile. Although she was alert and interactive, her psychomotor development was delayed, particularly gross motor skills, partly due to poor muscle bulk. Cranial CT was normal at 9 months of age. At 10 months, she suffered bronchiolitis due to respiratory syncytial virus leading to chronic liver failure, with hepatomegaly, ascites, hypoalbuminaemia and coagulopathy. The liver function improved subsequently but a further infection at the age of 12 months rapidly led to multiorgan failure and death.

**Family 3*. Patient 3*** was the 3^rd^ child of first cousin parents. She had two older sisters who were both fit and well. The family history revealed two relatives who had died in infancy due to liver disease (the mother’s sister’s two children whose parents were also consanguineous). This female baby was born at 38 weeks of gestational age with a birth weight of 2.38 kg (IUGR). During the immediate postnatal period, she had feeding problems but she was discharged on the 4^th^ day. Subsequently the results of the Guthrie cards showed raised tyrosine levels and she was admitted to the local hospital. Further investigations revealed abnormal liver function tests with coagulopathy and increased blood lactate values. She was discharged home on oral Vitamin K 1 mg with some improvement to her coagulopathy. Follow up revealed failure to thrive and a further admission at the age of 3 months. Blood tests showed a worsening coagulopathy and hypoglycaemia, which settled on continuous naso-gastric feeds. The coagulopathy was managed with daily Vitamin K, alternate FFP and cryoprecipitate infusions. She was found to be hypotonic with abnormal liver function tests and raised blood lactate. On abdominal ultrasound scan a distended gall bladder with some echogenic material consistent with biliary sludge was noted, but the liver, the spleen and the kidneys were normal. A brain MRI was normal and an ophthalmology review showed normal fundoscopy. Because of persistent coagulopathy, hypoglycaemia and liver dysfunction the patient was referred to a paediatric liver centre. On admission she was pale and mildly jaundiced with pedal oedema. Abdominal ultrasound showed a homogenous liver parenchyma with nodular margin, normal spleen and a small quantity of ascites. A liver biopsy could not be done because of the coagulopathy, but the patient underwent a muscle biopsy and bone marrow aspirate, together with a skin biopsy covered with fresh frozen plasma, cryoprecipitate and platelets. She was managed with vitamin supplements, IV antibiotics, anti-fungals and diuretics as well as albumin 20 % infusions to control the ascites. Because of persisting coagulopathy and increasing jaundice she underwent exchange transfusion. Following the results of the normal muscle mitochondrial respiratory chain analysis and with no evidence of clear neurological involvement, she was listed for liver transplant. Because of poor nutrition, parental nutrition was started and she required on-going support with clotting products and ascitic taps to manage her ascites. At the age of 4 months she was admitted to a paediatric intensive care unit for exchange transfusion. INR had increased to 7.46. She was re-admitted to the paediatric intensive care unit because of gradual deterioration with encephalopathy and she passed away at the age of 4 months.

**Family 4*. Patient 4*** is a girl who is the 5^th^ out of 6 children of consanguineous parents. She was born by normal delivery. There had been no initial problems and her growth and development was normal. Since then she has had some feeding problems needing calorie supplements and abnormal liver function tests but has not had any significant clinical issues with this to date. At the age of 5 years she presented with symptoms including unsteadiness and impaired co-ordination, she had been losing weight and was rather floppy. Her school work has not been affected. She has developed neurological problems with muscle weakness, failure to thrive, incoordination and ataxia showing a slow but progressive clinical deterioration. Investigation by ECG, EEG and cardiac ECHO was normal. On examination at the age of 5 yrs 10 months she was pale and thin. Cardiovascular and respiratory systems were normal. Neurological examination showed upper limb hypotonia, dysdiadochokinesis, impaired walking on tip toes, lower limb weakness and lower limb incoordination due to peripheral neuropathy affecting her legs.

***Patient 5*** was the 3^rd^ child. This baby girl jaundiced for two weeks after birth and and jaundice recurred at nine months of age at which time she was investigated. After this her growth slowed and liver function tests were intermittently abnormal. At the age of 2 years and 2 months she suddenly deteriorated, she became jaundiced with progressing weakness, distended abdomen and recurrent abdominal pain. She became odematous, drowsy and markedly hypotonic. She was started on the treatment protocol for acute liver failure (IV carnitine, N-acetyl cysteine, broad-spectrum antibiotics and antifungals). Her viral serology was positive for hepatitis A specific IgM. She continued to receive full support for liver failure with high doses of IV vitamin K. Liver ultrasound showed that her liver parenchyma was slightly heterogenous, and she had marked ascites in the abdomen and pelvis. At laparoscopy the liver surface appeared diffusely nodular with a picture of micro and macro nodular cirrhosis. As she was deemed to have severe progressive liver disease she was assessed for a liver transplant. Despite the apparent improvement of the biological markers and the stable clinical observations her general alertness and strength deteriorated. Neurological examination showed generalised hypotonia and hyporeflexia. Relentless progressive deterioration of muscle power necessitated nasogastric tube feeding. She also presented with a subcorneal opacity in her eyes which did not seem to be associated with a sensation of burning or pain. The ophthalmologist diagnosed a pericorneal marginal ulcer. She presented with acute, severe deterioration of liver function following an infection with hepatitis A. Progressive neurological deterioration with radiological signs of diffuse brain damage led to her death at the age of 4 years and 3 months.

***Patient 6*** was the 2^nd^ child in the family. He developed progressive jaundice at nine months of age. He deteriorated quickly and received a liver transplant at the age of 15 months. His hepatectomy suggested micronodular cirrhosis and end stage liver disease with no steatosis. He responded well to his transplant but presented with progressive neurological and renal symptoms leading to death at the age of 2.5 years.

**Family 5*. Patient 7*** was the first child of healthy first cousin parents. The family history was noteworthy because of liver disease in two members of the family and for premature deaths of two other infants. This baby presented at the age of 3.5 months with poor feeding, weight loss (growth on the 0.4^th^ centile), crying and vomiting. He was admitted to hospital where he was found to have hepatomegaly. He had lactic acidosis and acute liver failure with coagulopathy. A brain MRI was normal. Liver ultrasound showed an enlarged echogenic liver, but the spleen and kidneys were normal. Lactic acidosis and fatty infiltration in muscle suggested an underlying mitochondrial disorder. He was transferred to the liver unit in acute liver failure with encephalopathy, coaglulopathy, varices and upper gastrointestinal bleeding. The coagulopathy and cholestasis progressed in parallel throughout the course of the disease, with intermittent spontaneous improvements lasting 2 weeks until the final irreversible decline in liver function and death at the age of 4 months. With the exception of the residual changes related to the previously noted infarct, the brain magnetic resonance imaging (MRI) was considered normal, and proton magnetic resonance spectroscopy (^1^H-MRS) did not show an elevated lactate peak. The autopsy showed a single vessel thrombosis in brain without evidence of demyelination, and hepatic fatty infiltration.

***Patient 8***, a younger sister of Patient 7, was born at term weighing 3140 grams. Initially she presented with tachypnoea and hypoglycaemia (blood glucose 0.8 mmol/l). Laboratory investigations at 24 h of age revealed metabolic acidosis with persistent raised blood lactates. She was symptom-free at time of discharge at the age of four days. Due to the family history with liver disease, a cord blood sample was taken for genetic investigations. During follow-up she did not regain her birth weight and became jaundiced. On examination at the age of 1 month she was alert with a vigorous cry, but she was pale and jaundiced. Her abdomen was soft and there was no enlargement of her liver or spleen. The rest of the general examination was normal. She fed moderately well initially but was sleepy towards the end of the feed. Subsequently she was fed with high energy formula supplemented with vitamin K (1 mg orally daily) but failed to gain weight. She presented with progressive liver dysfunction leading to the death at the age of 7.5 months.

**Family 6*. Patient 9*** is a boy who was born at term, following uneventful pregnancy, to non consanguineous Caucasian parents. He was referred at 5 months with abnormal liver tests, failure to thrive and a marginally raised lactate on only one occasion. Full diagnostic work up revealed no underlying cause for his liver disease and he was managed conservatively. Over the next 15 months he developed cirrhosis and had a number of admissions for management of intermittent ascites. Skin biopsy showed normal pyruvate dehydrogenase activity. Initial muscle biopsy at the age of 28 months was within normal limits. Respiratory chain enzyme assays were equivocal with borderline complex I activity. At 3 years he had acute decompensation with coagulopathy and encephalopathy and he was assessed for liver transplantation. MRI brain was normal at this stage as was a repeat skin biopsy. Repeat muscle biopsy showed minor fatty changes and slightly reduced respiratory chain complex I and cytochrome oxidase (82% of normal). He subsequently underwent successful liver transplantation. Explanted liver showed micro-and macrovesicular steatosis, abundant mitochondria with mild pleomorphism and ‘fluffy’matrix. Cytochrome oxidase activity was 27% of normal. His subsequent course was uneventful until 4 years post liver transplantation when he developed abnormal transaminases due to presumed early chronic rejection. This resolved with increased immunosuppression. By 8 years of age his growth had slowed due to severe growth hormone deficiency and hypoparathyroidism. In addition he now required nasogastric tube feeding to support his nutritional intake as he was unable to eat adequate amounts of calories, though even optimal nutritional support did not improve his faltering growth. By 10 years of age he developed, initially slowly but subsequently rapidly, progressive mobility problems as a result of lower limb muscle tightness and contractures. Further investigations confirmed severe demyelinating peripheral neuropathy which has continued to progress over time. MRI brain at 11 years of age was normal. This patient is currently 11.5 yrs old. He liver graft functions well, on intensive nutritional support through gastrostomy. However he has a severe debilitating progressive demyelinating peripheral neuropathy.

**Family 7*. Patient 10*** was a boy, the eighth child to the non-consanguineous parents. He was born by normal delivery at term with uneventful pregnancy and no history of any medication during pregnancy. The birth weight was 3kg below the 5^th^ percentile. At the age of 2 months, he was admitted with a history of neonatal cholestatic jaundice, high lactic acidosis and diarrhoea. He has microcephaly, failure to thrive, hypotonia and hepatosplenomegaly. The family history was unremarkable. Laboratory investigations revealed abnormal liver function tests and raised blood lactates. His virological screening for Hepatitis B, Hepatitis C, CMV and EBV were negative. His metabolic workup for blood and urinalysis were not remarkable. High-resolution routine chromosomal analysis was normal. The echocardiogram was normal and the slit lamp eye exam showed retinal pigmentation. His brain MRI showed subcortical white matter changes. Histopathological studies on liver biopsy were abnormal (see Table 1). At the age of 4 months, he was readmitted to the hospital with intractable ascites. The patient also had severe failure to thrive despite a maximum calorie intake via nasogastric tube, hypotonia, progressive hepatosplenomegaly and deterioration of the liver synthetic function. He died at the age of 5 months.

**Family 8*. Patient 11*** was a girl born to consanguineous parents. Her two cousins had died due to uncharacterised liver disease. At the age of 2 months she presented with poor feeding, hypotonia and jaundice. She was found to have liver dysfunction, coagulopathy, and raised plasma lactate. At the age of 3.5 months liver biopsy showed non-specific changes and brain MRI was normal. She was subsequently lost to follow-up, but is known to have died between 7 and 16 months of age.

**Family 9**. ***Patient 12*** was referred at the age of 5 months with cholestatic jaundice and frequent hypoglycaemia that was first noted during the second month of life. She had been born at term, a normal spontaneous vaginal delivery with normal growth parameters (weight 3.9 kg, height 57cm, head circumference 37.7cm). Jaundice progressed with dark urine and pale stools associated with decreased feeding and failure to gain weight. Clinical examination revealed icteric sclerae, puffy eyes and light bronze-coloured skin. She was hypotonic with subtle facial dysmorphysim; bifrontal narrowing , synophrys, low-set and posteriorly rotated ears, migrognathia. The abdomen was distended, liver 5 cm below costal margin with prominent edge and firm consistency. The spleen was not palpable. Ophthalmologic examination was normal. Cardiac examination and echocardiography was normal. Abdominal US showed enlarged liver with diffuse fatty infiltration without any focal lesions. There was a small quantity of biliary sludge and of free intraperitoneal ascites. The size and echotexture of the spleen and kidneys were normal without any focal lesion. Spine x-ray did not reveal vertebral segmentation. Brain CT and MRI were unremarkable at 4 month of age. Blood acylcarnitines and amino acids were normal. Urine organic acid showed increased excretion of 4-hydroxyphenyllactic acid and 4-hydroxyphenylacetic acid. She continued to have hypoglycaemia and recurrent vomiting, fever and diarrhoea. Stool, urine and blood septic investigations revealed no pathogens. Initial biochemistry revealed a metabolic lactic acidosis. She needed frequent doses of intravenous hydrocortisone and dextrose because of ACTH deficiency. She was put on frequent feeding and blood glucose measurements to maintain normoglycaemia. She developed progressive abdominal distension causing breathing difficulties. She required frequent intravenous albumin, diuretics and paracentesis. She needed episodic blood product replacement, vitamin K injections for coagulapathy. Upon discharge home she was put on a mitochondrial cocktail, hydrocortisone, diuretics, calcium, vitamins and ursodeoxycholic acid. At 6 months of age, she developed severe episodes of hypoglycaemia and acidosis with chest infection. She became severely icteric with severe ascites, persistent lactic acidosis, hypoglycaemia and pulmonary oedema requiring intensive care. She died at the age of 8 months with respiratory haemorrhage, refractory anaemia, coagulopathy and thrombocytopaenia.

***Patient 13*** was born at term, a normal spontaneous vaginal delivery (birth weight 3.3 kg, length 52 cm and head circumference 34 cm, all below 3rd centile for age). She had jaundice, a mild metabolic acidosis and hyperlactataemia, although other biochemistry and liver transaminases were unremarkable. Her jaundice declined in two weeks. Urine organic acids measured on day 2 of life showed a high level of 4-hydroxyphenyllactic acid and 4-hydroxyphenylpyruvic acid. Tandem Mass spectrometry demonstrated slightly elevated tyrosine but she was discharged from hospital in good condition. Further evaluation at 5 months of age revealed motor delay, hypotonia with intact developmental milestones. She attended hospital because of recurrent vomiting following upper respiratory tract infection. She was found to have a distended abdomen with firm hepatomegaly.

**Family 10**. ***Patient 14*** is now a 10 month old Saudi male infant, born to consanguineous parents. He presented at aged 4 months with jaundice from the first week of life, failure to thrive, and developmental delay. In the family history, two siblings had died at 5 months and 11 months of age, due to undiagnosed liver disease. On physical examination, he was found to be hypotonic, jaundiced with enlarged liver 3 cm below costal margin, no splenomegaly. After admission he developed hypoglycaemia and needed glucose infusion. Extensive workup ruled out endocrine, metabolic, infectious, structural causes of cholestasis. Eye examination was normal. MRI brain and MR spectroscopy revealed no structural brain abnormality, no evidence of white or grey matter disease, and no evidence of changes suggestive of mitochondrial or metabolic changes. Currently, he is still alive at 10 months of age but has liver impairment.

**Family 11*. Patient 15*** is a daughter of consanguineous parents. She presented with conjugated jaundice and congenital hypothyroidism at the age of 10 weeks. Then she presented with failure to thrive, hypotonia, developmental delay, dysmorphism and raised blood lactate. Since 13 months of age she was lost from the follow-up as the family moved to another country. She died at the age of 3 years.

**Family 12*. Patient 16*** was a male baby, who presented with failure to gain weight, hepatomegaly, conjugated hyperbilirubinaemia, hypoglycaemia, hypotonia, and mild dysmorphic features. His parents are first cousins. He was admitted to hospital at the age of 2.5 months with poor feeding and weight loss. At the age of 3 months his weight was 2.8 kg and length was 55.5 cm. He had lactic acidosis, with recurrent hypoglycaemic episodes, faltering growth, generalised hypotonia, and conjugated hyperbilirubinaemia. Nasogastric feeds were started, complicated by recurrent vomiting. On examination he was floppy, lacking subcutaneous fat with hepatomegaly. During his stay liver function increasingly worsened with vitamin K-unresponsive coagulopathy and he became encephalopathic. The treatment was supportive, coagulopathy was corrected with repeated FFP transfusions. At the age of 4 months he had an episode of haematemesis suggesting pulmonary haemorrhage. Brain MRI revealed some subtle changes within the deep white matter of the posterior parietal lobe on T2 images. In these regions there were slightly high signal on the diffusion images. Lactate peak was detected within the basal ganglia and the white matter on spectroscopy. He died following cardiorespiratory arrest.

***Patient 17*** is a 5 month old baby girl. Prenatal diagnosis at 17 weeks gestation showed that she had the same *MPV17* genotype as patient 16. Nevertheless, the parents opted to continue the pregnancy. The baby was in good condition at birth, and a fibroblast line was set up from umbilical cord. This showed mosaic depletion (figure 2). Gamma glutamyl transferase was raised at 6 weeks of age. She developed progressive liver failure from 4 months of age and is now deteriorating rapidly.
