# Supplementary material for: Clinical, biochemical, cellular and molecular characterization of mitochondrial DNA depletion syndrome due to novel mutations in the MPV17 gene
Source: Eur J Hum Genet. 2013 May 29;22(2):184–91. doi: 10.1038/ejhg.2013.112 (PMC3895632; doi:10.1038/ejhg.2013.112)

Supporting material 2

Quantitative confirmation of mosaic mtDNA depletion identified by light microscopy using independent "blinded" microscopist and INCell1000. These data show that qualitative analysis by PicoGreen staining is both sensitive and specific.

	Qualitative analysis of fibroblasts	Microscopy 1			Microscopy 2			INCell1000 analysis			QPCR
Patient		Cells counted	Mean % depleted	Mean % Rho°	Cells counted	Mean % Rho°	Std. Dev Mean	Cells counted	Mean nucleoid count	Std. Error Mean	MtDNA content %
2	Normal	599	12	0.0	910	0	0.0	819	139	3.4	103
4	Normal/ minor changes	596	12	0.0	1426	<1	0.2	1255	80	1.6	43
8	Normal/ minor changes	410	<1	<1	692	0	0.0	401	326	22.3	149
9	Normal	640	5	<1	1124	0	0.0	1099	97	1.9	59
11	Mosaic depletion	725	22	5.2	1270	6	2.2	1245	63	1.0	33
15	Mosaic depletion	1054	30	12.0	1526	7	1.4	1400	68	1.1	100
17	Mosaic depletion *	29	10	14							78
Control	Normal	651	2	<1	918	0	0.0	1025	77	1.5	72

* Patient 17's cultures are no longer viable, so quantititation was carried out on existing images

The average number of nucleoids detected per cell using automated IN Cell 1000 is proportional to mtDNA copy number from Q PCR

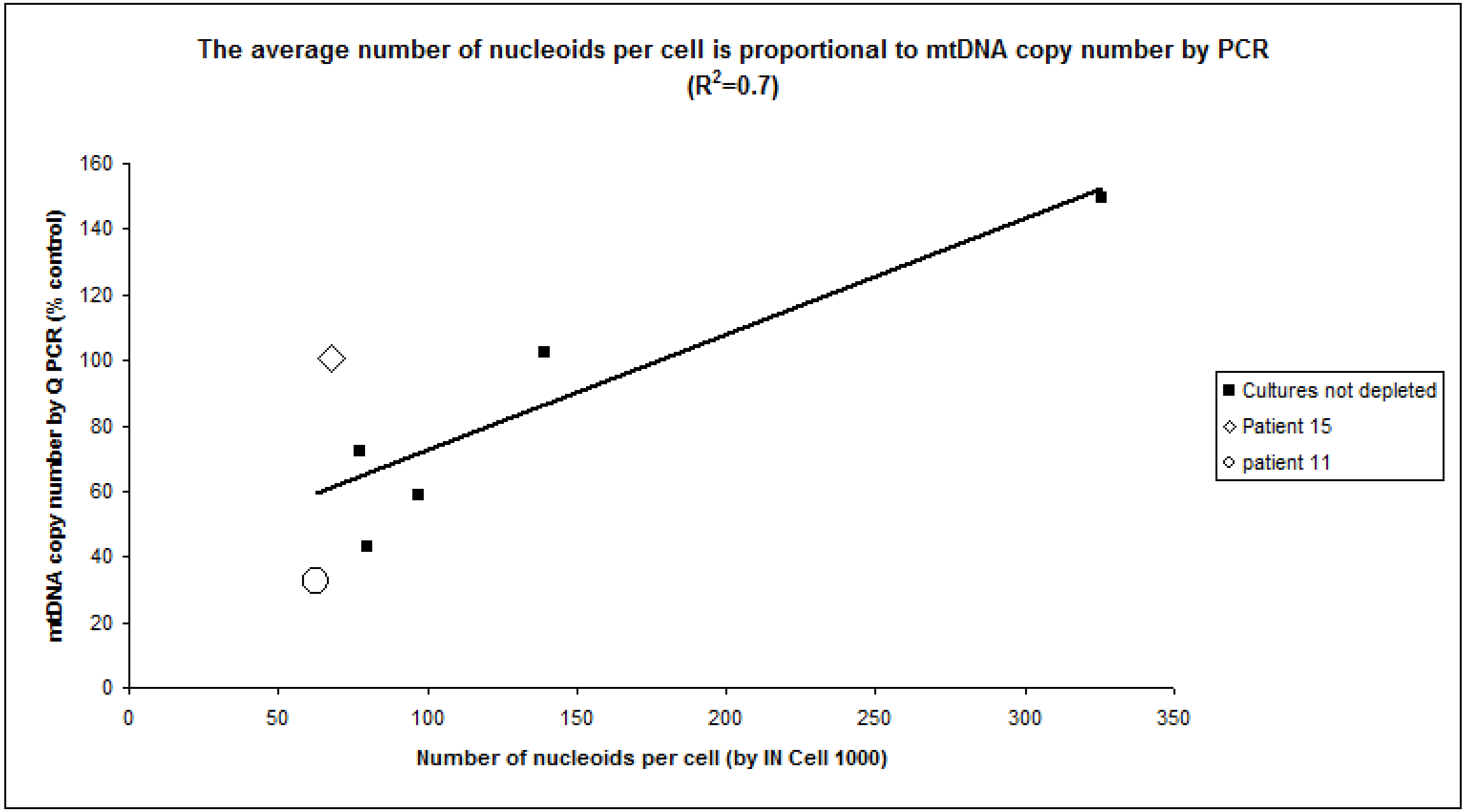

Supplement: Supplementary Material 2 [file ejhg2013112x3.pdf]
